# Joint Associations of Actual Age and Genetically Determined Age at Menarche With Risk of Mortality

**DOI:** 10.1001/jamanetworkopen.2021.15297

**Published:** 2021-06-30

**Authors:** Zhaoxia Liang, Hao Ma, Qiying Song, Dianjianyi Sun, Tao Zhou, Yoriko Heianza, Danqing Chen, Lu Qi

**Affiliations:** 1 Department of Epidemiology, School of Public Health and Tropical Medicine, Tulane University, New Orleans, Louisiana; 2 Department of Obstetrics and Gynecology, Women’s Hospital, Zhejiang University School of Medicine, Hangzhou, Zhejiang Province, China; 3 Department of Maternal and Child Health, School of Public Health, Peking University, Beijing, China; 4 Department of Epidemiology and Biostatistics, School of Public Health, Peking University, Beijing, China; 5 Department of Nutrition, Harvard T.H. Chan School of Public Health, Boston, Massachusetts; 6 Channing Division of Network Medicine, Department of Medicine, Brigham and Women’s Hospital and Harvard Medical School, Boston, Massachusetts

## Abstract

**Importance:**

Previous studies have shown an association between actual age at menarche and risk of all-cause mortality; however, the results are inconsistent, and no study has analyzed the joint associations between genetic susceptibility and actual age at menarche with the risk of mortality in prospective cohorts.

**Objectives:**

To investigate joint associations of actual age and genetically determined age at menarche with risk of all-cause mortality.

**Design, Setting, and Participants:**

This prospective cohort study was conducted using data from the UK Biobank population across the United Kingdom from March 13, 2006, to October 1, 2010. A total of 264 546 women aged between 39 and 71 years with actual menarcheal age were included in this study; 246 676 of these women had genetic data available. Actual age at menarche was obtained from the touchscreen questionnaire at recruitment from 2006 to 2010. Genetically determined age at menarche was assessed by a genetic risk score. Statistical analysis was performed from August 22 to December 12, 2019.

**Exposure:**

Age at menarche.

**Main Outcomes and Measures:**

A multivariable Cox proportional hazards regression model was used to assess associations of actual or genetically determined age at menarche with risk of all-cause mortality.

**Results:**

The mean (SD) age of the study population at baseline was 56.4 (8.0) years, and the mean (SD) age at menarche included in the analyses was 13.0 (1.6) years. During a median of 9.0 years (range, 8.3-9.7 years) of follow-up, 7761 deaths were documented among the women with actual age at menarche, and 7054 deaths were documented among the women with genetically determined age at menarche. Both the actual age at menarche and the genetically determined age at menarche showed a U-shaped association with the risk of all-cause mortality (lowest actual age [<12 years] vs reference age [15 years]: hazard ratio [HR], 1.16 [95% CI, 1.07-1.26]; highest actual age [≥16 years] vs reference age [15 years]: HR, 1.17 [95% CI, 1.05-1.31]; *P* < .001 for quadratic trend; genetic risk score [GRS] of 1 vs reference score [GRS of 4]: HR, 1.10 [95% CI, 1.01-1.19; GRS of 6 vs reference score [GRS of 4]: HR, 1.09 [95% CI, 1.00-1.18]; *P* = .03 for quadratic trend). Significant interactions were also found between actual age at menarche and genetically determined age at menarche with all-cause mortality (HR of mortality associated with age of menarche <12 year was 1.24 [95% CI, 1.10-1.40] in the GRS of 1 group and 1.44 [95% CI, 1.21-1.72] in the GRS of 6 group; *P* = .001 for interaction). Women with mismatch of actual age and genetically determined age at menarche had the highest mortality risks; participants with the lowest genetic risk score and the highest age at menarche had an HR of 2.12 (95% CI, 1.58-2.83), and participants with the highest GRS and the lowest age at menarche had an HR of 1.44 (95% CI, 1.21-1.72).

**Conclusions and Relevance:**

The results suggest that both actual age and genetically determined age at menarche exhibit U-shaped associations with all-cause mortality. Women with mismatch of actual age and genetically determined age at menarche may have the highest risk of all-cause mortality.

## Key Points

**Question:**

What are the joint associations of actual age and genetically determined age at menarche with risk of all-cause mortality?

**Findings:**

This cohort study found that that both actual age and genetically determined age at menarche showed similar U-shaped associations with risk of all-cause mortality in a large group of women. The study also found a significant interaction between actual and genetically determined age at menarche with all-cause mortality; women with mismatch of actual age and genetically determined age at menarche had the highest risk of all-cause mortality.

**Meaning:**

This study suggests that women with a mismatch of actual age and genetically determined age at menarche may have a significantly higher risk of all-cause mortality than women whose actual age and genetically determined age at menarche are matched.

## Teaser

This cohort study uses data from the UK Biobank to investigate joint associations of actual age and genetically determined age at menarche with risk of all-cause mortality.

## Introduction

Menarche signifies the beginning of the reproductive period, which marks dramatic physiological changes associated with reproductive and various biological functions in female individuals.^1^ Age at menarche shows considerable variability, which has been associated with chronic diseases such as cardiovascular disease, metabolic syndrome, and cancers.^2^

Several epidemiologic studies have analyzed the association between age at menarche and all-cause mortality.^3,4,5,6,7,8,9^ However, the findings are highly inconsistent; some studies reported a linear inverse association between age at menarche and all-cause mortality,^3,7^ whereas others found a U-shaped association^5,6,9^ or a null association.^10^ However, to our knowledge, little is known about the potential factors associated with the heterogeneous risk of all-cause mortality observed in the populations studied.

In the past decade, genome-wide association studies have identified a group of genetic variants associated with age at menarche.^11^ We assumed that genetic predisposition might modulate the association between age at menarche and mortality. Actual age at menarche is associated with environmental factors, so analyses of joint associations of the actual age at menarche and genetically determined age at menarche may reflect the interactive associations of environment and genetic factors with health outcomes. However, to our knowledge, no study has analyzed the joint associations of actual age and genetically determined age at menarche with risk of all-cause mortality.

Therefore, by taking advantage of comprehensive information collected from a large prospective cohort study, the UK Biobank, we investigated the associations of actual age at menarche and genetically determined age at menarche, evaluated as a genetic risk score (GRS), with the risk of all-cause mortality among middle-age and older women (range, 39-71 years). In particular, we assessed the joint associations of actual age and genetically determined age at menarche with mortality risk.

## Methods

### Study Population

The UK Biobank is a large prospective cohort study, recruiting more than 500 000 participants aged 37 to 73 years during the period from 2006 to 2010 from across the United Kingdom. The details of the study design have been reported in previous publications.^12,13^ In the present study, we included 264 546 women aged between 39 and 71 years who had completed data on age at menarche at baseline. After excluding participants with sex discordance or high missingness of variables or heterozygosity and those who were genetically related to others in the study, genetic data were available from 246 676 of the 264 546 participants. The UK Biobank study and the current study were approved by the National Health Service National Research Ethics Service and institutional review board of Tulane University Health Sciences Center. Written informed consent was obtained from all participants. The study follows the Strengthening the Reporting of Observational Studies in Epidemiology (STROBE) reporting guideline for cohort studies.

### Exposure Assessment

Data on age at menarche were obtained from a touchscreen questionnaire at recruitment during the period from 2006 to 2010. Participants were asked to report “How old were you when your periods started?” Answers between 5 and 25 years were accepted. If the answers were younger than 6 years or older than 20 years, the participants were asked to confirm. If women reported “do not know” or “prefer not to answer,” the answer was coded as missing. In this analysis, age at menarche was classified into 6 categories: 11 years or younger, 12 years, 13 years, 14 years, 15 years, and 16 years or older. To assess the validity of information on the age at menarche, 2 surveys (the first visit was completed from 2012 to 2013 and the second visit was conducted in 2014 or later) were conducted with the same questionnaire. Age at menarche reported at baseline was highly correlated with the reported age in the first survey (Spearman ρ = 0.89) and the second survey (Spearman ρ = 0.86).

### Genotyping and GRSs

Detailed information on genotyping and imputation of the UK Biobank has been published previously.^14^ In the present study, we calculated the GRSs for age at menarche based on 340 single-nucleotide variations (SNVs) reported in a recent genome-wide association study that included UK and other populations.^11^ The details about the selected SNVs are provided in the eTable in the Supplement.

We used a weighted method to calculate the GRS on the basis of the 340 SNVs. Each SNV was recoded as 0, 1, or 2 according to the number of risk alleles, and each SNV was weighted by its relative effect size (β coefficient) on age at menarche obtained from the previous genome-wide association study.^11^ We calculated the GRS by using the equation GRS = (β1 × SNV1 + β2 × SNV2 + … + βXX × SNV340) × (340/sum of the β coefficients), where SNVi is the risk allele number of each SNV.^15^ The GRS ranges from 0 to 359.7. A higher GRS indicated late age at menarche, and a lower GRS indicated earlier age at menarche.

If women’s actual age and genetically determined age at menarche were inconsistent, such as those with the lowest GRS but the highest actual age at menarche, or those with the highest GRS but the lowest actual age at menarche, they were defined as “mismatched.”

### Ascertainment of Outcomes

The date of death was obtained from the death certificate in the National Health Service Information Centre (England and Wales) and the National Health Service Central Register Scotland (Scotland).^16^ In this analysis, mortality data were available up to February 14, 2018. Therefore, mortality analysis was censored at this date, the date of death, or the follow-up termination date, whichever occurred first.

### Assessment of Covariates

Participants provided information on sociodemographic characteristics, lifestyle, reproductive factors, medical history, and early-life factors through touchscreen questionnaires or verbal interview and completed a series of physical measurements and biological sample collection. Sociodemographic characteristics at baseline included age, race/ethnicity (White, mixed, Asian, Black, others, or missing), assessment center, and Townsend deprivation index (comprehensive measure of deprivation based on unemployment, nonownership of a car, nonownership of a home, and household overcrowding, reflecting socioeconomic status; a higher Townsend deprivation index implies a greater degree of deprivation).

Information about lifestyles included smoking status (never, former, current, or missing), alcohol drinking status (never, former, current, or missing), physical activity, and diet. According to the International Physical Activity Questionnaire scoring protocol for the short form,^17^ physical activity was categorized into low, moderate, and high levels. A healthy diet score was evaluated by servings of vegetables (≥3 per day), fruits (≥3 per day), fish (≥2 per week), and red meat (<1.5 per week); 1 point was given for each favorable diet factor, and the total diet score ranges from 0 to 4. Data on reproductive factors included menopause status (yes, no, or missing) and parity (0, 1, 2, 3, ≥4, or missing). Data on history of cancer, cardiovascular disease (CVD; heart attack, angina, or stroke), and diabetes were obtained from the diagnosis from physicians. Hypertension was defined as self-reported systolic blood pressure of 140 mm Hg or more or diastolic blood pressure of 90 mm Hg or more or use of antihypertensive medications. A high cholesterol level was defined as self-reported or use of lipid-lowering treatment. Early life factors included birth weight, which was classified into categories of underweight (<2.5 kg), normal weight (2.5-4 kg), and macrosomia (≥4 kg) or missing.

Standing height was measured by a Seca 202 stadiometer. Weight was measured by the Tanita BC-418 MA body composition analyzer, accurate to the nearest 0.1 kg. Body mass index (BMI) was calculated as weight in kilograms divided by height in meters squared.

### Statistical Analysis

Statistical analysis was performed from August 22 to December 12, 2019. The associations of age at menarche (or GRS of age at menarche) with all-cause mortality were estimated by multivariable Cox proportional hazards regression models with the number of years of follow-up as the time metric, and the results were reported as hazard ratios (HRs) and 95% CIs. Covariates in the multivariate-adjusted model included age, race/ethnicity, Townsend deprivation index, smoking status, alcohol status, physical activity, menopause status, parity, BMI, healthy diet score, birth weight, assessment center, and history of cancer, CVD, diabetes, hypertension, and high cholesterol. For analyses of genetic data, we further adjusted for the first 5 genetic principal components and genotyping array. We considered the corresponding lowest HR group as the reference. The proportional hazards assumption was tested and satisfied by the Schoenfeld residuals method. We coded the missing data as mean values for continuous variables and with another category for categorical variables.

We conducted stratified analyses to evaluate potential modification effects by the following factors: smoking status (never or ever), BMI (<30 or ≥30), physical activity (≥600 or <600 metabolic equivalent minutes per week), alcohol consumption status (never or ever), parity (0 or ≥1), menopause status (yes or no), age (≤56 or >56 years), healthy diet score (≤2 or >2), and birth weight (<2.5 or ≥2.5 kg). To evaluate the interaction between age at menarche and GRS of age at menarche with the risk of all-cause mortality, multiplicative interaction was evaluated by adding interaction terms to the Cox proportional hazards regression models. Joint associations of age at menarche and genetically determined age at menarche with all-cause mortality were also tested. During analysis of the joint associations, we combined the second and third groups into 1 group, and the fourth and fifth groups into 1 group. Therefore, age at menarche and GRS of age at menarche were also divided into 4 groups, leading to a total of 16 subgroups. In sensitivity analyses, we repeated the main analyses for White participants.

All statistical analyses were performed using SAS, version 9.4 (SAS Institute Inc). All statistical tests were 2-sided, and statistical significance was set at a 2-tailed *P* < .05.

## Results

### Baseline Characteristics of Participants

The Table shows the baseline characteristics of the study participants according to age at menarche. Overall, 264 546 women in the study population reported their age at menarche. The mean (SD) age of our study population at baseline was 56.4 (8.0) years, and the mean (SD) age at menarche was 13.0 (1.6) years. The proportion of women who reached menarche at younger than 12 years was 20.1% (n = 53 058), the proportion of women who reached menarche at 12 years was 18.9% (n = 50 117), the proportion of women who reached menarche at 13 years was 24.4% (n = 64 513), the proportion of women who reached menarche at 14 years was 19.7% (n = 52 211), the proportion of women who reached menarche at 15 years was 11.0% (n = 29 058), and the proportion of women who reached menarche at 16 years or older was 5.9% (n = 15 589).

**Table.** Baseline Characteristics of UK Biobank Participants by Age at Menarche

Baseline characteristic	Age at menarche (in completed full-year intervals), y^a^					
	<12 (n = 53 058)	12 (n = 50 117)	13 (n = 64 513)	14 (n = 52 211)	15 (n = 29 058)	≥16 (n = 15 589)
Age, mean (SD), y	56.7 (7.7)	56.3 (7.9)	56.0 (8.0)	56.5 (8.1)	56.6 (8.1)	56.0 (8.1)
Race/ethnicity, No. (%)						
White European	50 445 (95.1)	47 418 (94.6)	61 201 (94.9)	49 616 (95.0)	27 355 (94.1)	14 091 (90.4)
Mixed	399 (0.8)	342 (0.7)	425 (0.7)	311 (0.6)	181 (0.6)	118 (0.8)
Asian	844 (1.6)	971 (1.9)	1227 (1.9)	1017 (1.9)	608 (2.1)	547 (3.5)
Black	754 (1.4)	778 (1.6)	915 (1.4)	726 (1.4)	584 (2.0)	549 (3.5)
Other	448 (0.8)	483 (1.0)	582 (0.9)	427 (0.8)	278 (1.0)	237 (1.5)
BMI, mean (SD)	28.57 (5.7)	27.35 (5.2)	26.71 (4.9)	26.50 (4.8)	26.37 (4.8)	26.22 (4.9)
Current smoker, No. (%)	4866 (9.2)	4083 (8.1)	5292 (8.2)	4669 (8.9)	2940 (10.1)	1796 (11.5)
Current alcohol consumption, No. (%)	47 731 (90.0)	45 729 (91.2)	59 003 (91.5)	47 436 (90.9)	25 989 (89.4)	13 571 (87.1)
Townsend deprivation index, mean (SD)	−1.3 (3.0)	−1.5 (3.0)	−1.4 (3.0)	−1.4 (3.0)	−1.2 (3.1)	−0.9 (3.3)
Physical activity level, No. (%)						
Low	29 992 (56.5)	28 357 (56.6)	36 033 (55.9)	28 764 (55.1)	15 839 (54.5)	8465 (54.3)
Moderate	20 680 (39.0)	19 693 (39.3)	25 773 (40.0)	21 119 (40.4)	11 800 (40.6)	6208 (39.8)
High	2386 (4.5)	2067 (4.1)	2707 (4.2)	2328 (4.5)	1419 (4.9)	916 (5.9)
Healthy diet score, mean (SD)	2.0 (1.1)	1.9 (1.1)	1.9 (1.1)	1.8 (1.1)	1.8 (1.1)	1.9 (1.1)
Postmenopausal, No. (%)	10 784 (20.3)	11 641 (23.2)	16 211 (25.1)	12 572 (24.1)	6884 (23.7)	3925 (25.2)
Parity, mean (SD)	2.8 (1.1)	2.7 (1.1)	2.8 (1.1)	2.8 (1.1)	2.9 (1.1)	2.8 (1.2)
Birth weight, mean (SD), kg	3.2 (0.7)	3.2 (0.6)	3.3 (0.6)	3.3 (0.6)	3.2 (0.7)	3.2 (0.7)
History of disease, No. (%)						
Cancer	5235 (9.9)	4700 (9.4)	5633 (8.7)	4699 (9.0)	2538 (8.7)	1312 (8.4)
CVD	2218 (4.2)	1555 (3.1)	1863 (2.9)	1739 (3.3)	1123 (3.9)	649 (4.2)
Diabetes	2699 (5.1)	1911 (3.8)	2093 (3.2)	1713 (3.3)	978 (3.4)	637 (4.1)
Hypertension	28 538 (53.79)	24 884 (49.65)	29 982 (46.47)	24 693 (47.29)	13 651 (46.98)	7137 (45.78)
High cholesterol	8555 (16.1)	6982 (13.9)	8031 (12.4)	6949 (13.3)	4146 (14.3)	2207 (14.2)
GRS of age at menarche						
No.	41 097	41 120	41 096	41 125	41 120	41 118
Mean (SD)	309.0 (10.5)	311.4 (10.4)	313.5 (10.4)	315.2 (10.4)	316.9 (10.5)	318.6 (10.6)

Abbreviations: BMI, body mass index (calculated as weight in kilograms divided by height in meters squared); CVD, cardiovascular disease; GRS, genetic risk score.

^a^ Missing data were coded as mean values for continuous variables, with another category for categorical variables.

Compared with women with late menarche, women with earlier menarche had a higher BMI and lower Townsend deprivation index. Women with earlier or later menarche were more likely than those with a menarcheal age of 12 to 15 years to have a history of CVD or diabetes or to be current smokers. Women with earlier menarche had a higher likelihood of receiving a diagnosis of (or reporting to investigators that they had) cancer, hypertension, and hypercholesterolemia at baseline than those with late menarche. Women with late menarche were more likely to exercise vigorously and were more likely to be postmenopausal at baseline. We also observed that women with late age at menarche had a higher GRS of age at menarche.

### Association Between Age at Menarche and All-Cause Mortality

**Figure 1.**
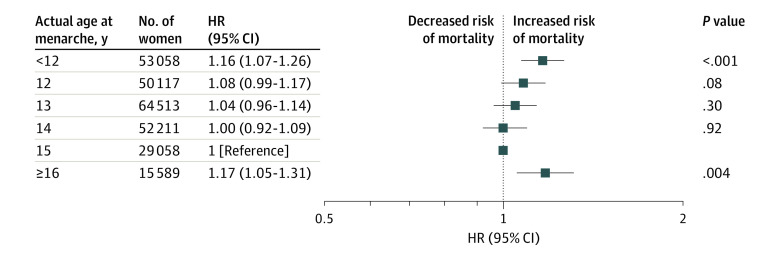
Multivariate Adjusted Risk of Mortality Associated With Women’s Actual Age at Menarche Analyses are adjusted for age, race/ethnicity, Townsend deprivation index, smoking status, alcohol consumption status, physical activity, menopause status, parity, body mass index, healthy diet score, birth weight, assessment center, and history of cancer, diabetes, cardiovascular disease, hypertension, and high cholesterol level. HR indicates hazard ratio.

During a median of 9.0 years (range, 8.3-9.7 years) of follow-up, we documented 7761 incident cases of all-cause mortality among the women with actual age at menarche and 7054 incident cases of all-cause mortality among the women with genetically determined age at menarche. In the age-adjusted analyses, we found a U-shaped association between age at menarche and all-cause mortality (lowest actual age [<12 years] vs reference age [15 years]: HR, 1.21 [95% CI, 1.12-1.32]; highest actual age [≥16 years] vs reference age [15 years]: HR, 1.21 [95% CI, 1.08-1.35]; *P* < .001 for quadratic trend). After further adjustment for race/ethnicity, Townsend deprivation index, smoking status, alcohol consumption status, physical activity, menopause status, parity, BMI, healthy diet score, birth weight, assessment center, and history of cancer, CVD, diabetes, hypertension, and high cholesterol, the U-shaped association remained significant (lowest actual age [<12 years] vs reference age [15 years]: HR, 1.16 [95% CI, 1.07-1.26]; highest actual age [≥16 years] vs reference age [15 years]: HR, 1.17 [95% CI, 1.05-1.31]; *P* < .001 for quadratic trend) (Figure 1). Women with menarche at 15 years of age had the lowest risk. Compared with this group, the HR associated with all-cause mortality increased significantly for women with either earlier or later ages at menarche. The highest risk for all-cause mortality was observed among women with menarche at younger than 12 years (HR, 1.16; 95% CI, 1.07-1.26) and at 16 years or older (HR, 1.17; 95% CI, 1.05-1.31).

**Figure 2.**
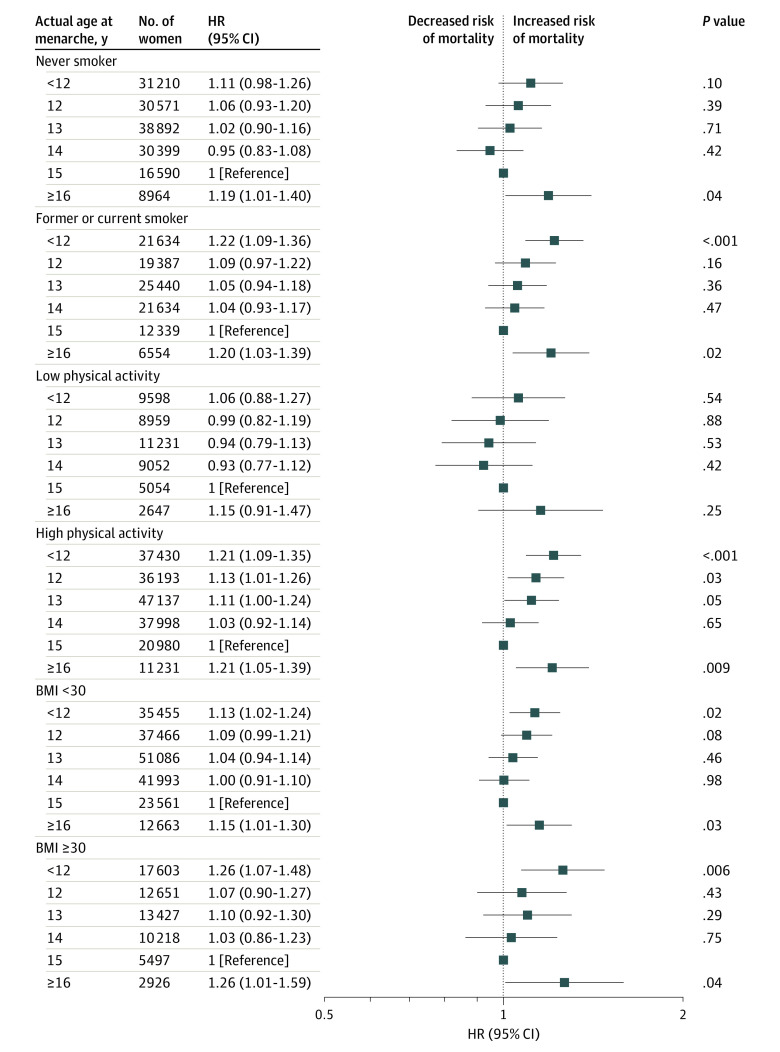
Multivariate Adjusted Risk of Mortality Associated With Actual Age at Menarche Stratified by Smoking Status and Physical Activity Analyses are adjusted for age, race/ethnicity, Townsend deprivation index, smoking status (except stratifying by smoker), alcohol consumption status, physical activity (except stratifying by physical activity), menopause status, parity, body mass index (BMI; calculated as weight in kilograms divided by height in meters squared), healthy diet score, birth weight, assessment center, and history of cancer, diabetes, cardiovascular disease, hypertension, and high cholesterol level. HR indicates hazard ratio.

We did not observe significant interactions between age at menarche and potential risk factors including age, smoking, alcohol consumption, physical activity, parity, menopause status, BMI, healthy diet score, or birth weight with risk of all-cause mortality. Figure 2 shows the association between age at menarche and all-cause mortality stratified by potential modifiable risk factors such as smoking, BMI, and physical activity.

### Joint Associations of Actual Age and Genetically Determined Age at Menarche With Risk of All-Cause Mortality

**Figure 3.**
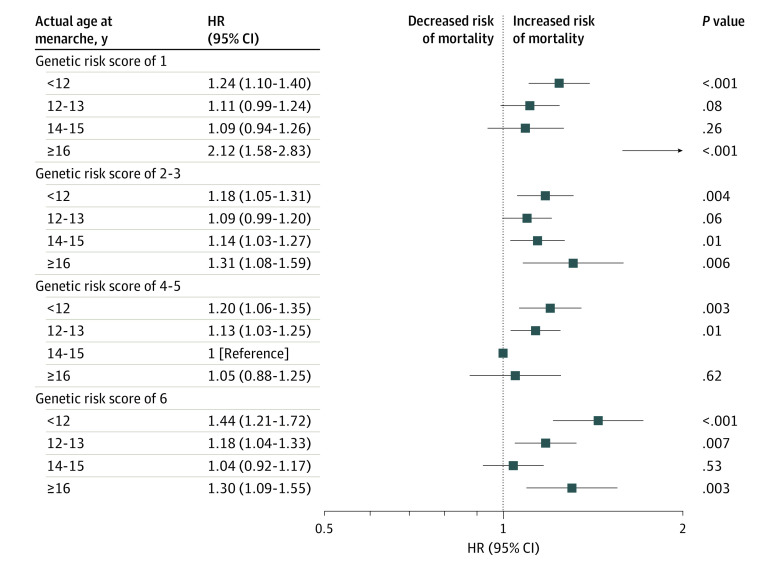
Interaction Between Actual Age and Genetically Determined Age at Menarche With Mortality Analyses are adjusted for age; race/ethnicity; Townsend deprivation index; smoking status; alcohol consumption status; physical activity; menopause status; parity; body mass index; healthy diet score; birth weight; assessment center; history of cancer, diabetes, cardiovascular disease, hypertension, and high cholesterol level; the first 5 genetic principal components; and genotyping array. HR indicates hazard ratio.

Similar to the results of actual age at menarche, we found a U-shaped association of GRS of age at menarche with risk of all-cause mortality in the fully adjusted model (GRS of 1 vs reference score [GRS of 4]: HR, 1.10 [95% CI, 1.01-1.19; GRS of 6 vs reference score [GRS of 4]: HR, 1.09 [95% CI, 1.00-1.18]; *P* = .03 for quadratic trend) (eFigure 1 in the Supplement). We further assessed the interactions between age at menarche and GRS of age at menarche with all-cause mortality. In age-adjusted and fully adjusted analyses, age at menarche significantly interacted with GRS of all-cause mortality (HR of mortality associated with age of menarche <12 year was 1.24 [95% CI, 1.10-1.40] in the GRS of 1 group and 1.44 [95% CI, 1.21-1.72] in the GRS of 6 group; *P* = .001 for interaction). In the joint analysis, we found that participants with the lowest GRS and the highest age at menarche had the highest HR for all-cause mortality (2.12; 95% CI, 1.58-2.83; Figure 3), followed by those with the highest GRS and the lowest age at menarche (HR, 1.44; 95% CI, 1.21-1.72; Figure 3).

### Sensitivity Analyses

In the sensitivity analyses, when we included only White women, the associations between age at menarche and all-cause mortality outcomes were still significant (lowest actual age [<12 years] vs reference age [15 years]: HR, 1.16 [95% CI, 1.06-1.26]; highest actual age [≥16 years] vs reference age [15 years]: HR, 1.19 [95% CI, 1.06-1.33]; *P* < .001 for quadratic trend; eFigure 2 in the Supplement). The U-shaped association between GRS of age at menarche and all-cause mortality also did not change appreciably (GRS of 1 vs reference score [GRS of 4]: HR, 1.10 [95% CI, 1.01-1.19; GRS of 6 vs reference score [GRS of 4]: HR, 1.09 [95% CI, 1.00-1.18]; *P* = .02 for quadratic trend; eFigure 3 in the Supplement). In addition, interactions between age at menarche and GRS of age at menarche with all-cause mortality also remained significant (HR of mortality associated with age of menarche <12 year was 1.23 [95% CI, 1.09-1.39] in the GRS of 1 group and 1.46 [95% CI, 1.22-1.74] in the GRS of 6 group; *P* < .001 for interaction; eFigure 4 in the Supplement).

## Discussion

In this large prospective cohort study, we confirmed that actual age at menarche showed a U-shaped association with risk of all-cause mortality, with the lowest risk of all-cause mortality for menarche at 15 years and the highest risk of all-cause mortality for menarche at younger than 12 years or 16 years or older. In addition, we observed a significant interaction between actual age and genetically determined age at menarche with all-cause mortality: women with mismatch of actual age and genetically determined age at menarche showed the highest risk of all-cause mortality.

The association between early age at menarche and higher risk of mortality has been well established in previous studies.^2,3,4,7^ However, the association between late menarche and risk of all-cause mortality is still unclear, which might be owing to the limited statistical power in those studies for participants with late age at menarche (≥16 years). In this study, we took advantage of the large sample size of the UK Biobank and observed a U-shaped association between age at menarche and risk of all-cause mortality, in which both early age at menarche and late age at menarche were significantly associated with an increased risk of all-cause mortality. In accordance with our results, the U-shaped association between age at menarche and risk of all-cause mortality were also observed in 2 previous studies and 2 meta-analyses,^5,6,8,9^ whereas the HRs for participants with late age at menarche did not reach statistical significance.

We also found that the genetically determined age at menarche was significantly associated with risk of all-cause mortality. Age at menarche is associated with genetic makeup,^18,19^ and the heritability for age at menarche estimated from family and twin studies ranged from 53% to 74%.^20^ In our study, we found that the GRS was strongly associated with actual age at menarche (ie, the late age at menarche with the higher GRS of age at menarche). Our analyses of the association between the genetically determined age at menarche and mortality further confirmed the U-shaped association, and the consistent results at the genetic level ensured the robustness of our findings.

The mechanisms underlying the observed U-shaped associations of age at menarche with risk of all-cause mortality remained unclear. Previous studies have shown the association between early age at menarche and increased risks of death-causing diseases, including cancer,^21,22,23,24^ CVD,^25,26,27,28^ diabetes,^29,30,31,32^ hypertension,^25,33,34^ and hypercholesterolemia,^3^ lending support to our findings. These previous studies suggest that early age at menarche may increase the risk of mortality through various mechanisms. Late age at menarche has also been associated with an elevated risk of CVD mortality^26^ and death-related risk factors such as smoking^35^ and malnutrition,^36^ both of which might partially explain the observations in our study.

A novel finding of the present study was the interaction of actual age at menarche and genetically determined age at menarche with all-cause mortality. We found that the associations between actual age at menarche and mortality differed according to the genetic predispositions. In addition, we found that women with the lowest genetically determined age at menarche and the highest actual age at menarche exhibited the highest risk of mortality, followed by women with the highest genetically determined of age at menarche and the lowest actual age at menarche. These results suggest that women with mismatch of genetic predisposition and actual age at menarche may have a higher mortality risk than women whose genetic predisposition and actual age at menarche are matched. The potential mechanisms have yet to be clarified. Previous studies have shown that actual age at menarche is associated with environmental factors, such as diet and lifestyle, among others.^37,38^ Therefore, women with a mismatch of genetic and actual age at menarche are likely those who are more subject to the adverse effect of environmental factors, and therefore they are more likely to have a higher risk of premature death. Our results suggested that the elevated risk associated with the mismatch could be reduced by dietary and lifestyle modifications. Further studies are warranted to investigate the pathophysiological alterations caused by such a mismatch, which may causally lead to an increased risk of death.

### Strengths and Limitations

This study has some strengths. To our knowledge, the present study is the first to explore the joint associations of actual age and genetically determined age at menarche with all-cause mortality. The major strengths of this study include the large sample size, the prospective design, and the lifestyle data, demographic characteristics, and detailed genetic and clinical information found to be associated with age at menarche.^12^

There are also several potential limitations. First, we used self-recalled information of age at menarche. However, previous studies have demonstrated a high correlation between age at menarche by recall in middle age and the validated data.^39,40^ Although recall bias was inevitable in this study, correlations were observed between baseline information and the information from 2 surveys, suggesting the validity of the data. Second, it has recently been shown that birth weight and childhood BMI are associated with age at menarche.^41^ Although data on childhood BMI were not available in our study, there is an association between BMI in childhood and adult BMI,^42,43^ and we have adjusted for birth weight and BMI in adulthood in our analyses.

## Conclusions

In this study, we found that actual age and genetically determined age at menarche showed similar U-shaped associations with risk of all-cause mortality in a large cohort of women. In addition, we found a significant interaction between actual age and genetically determined age at menarche with mortality risk; women with mismatched actual age and genetically determined age at menarche had a higher risk of mortality than women whose actual age and genetically determined age at menarche were matched. Further studies are needed to validate our findings and illustrate the potential mechanisms.

## Notes

Supplement. **eTable.** Characteristics of Genetic Variants Associated With Age at Menarche in the UK Biobank **eFigure 1.** Multivariate Adjusted Hazard Ratio (95% CI) for Mortality Associated With Women’s Genetically Determined Age at Menarche **eFigure 2.** Multivariate Adjusted Hazard Ratio (95% CI) for Mortality Associated With White Women’s Actual Age at Menarche **eFigure 3.** Multivariate Adjusted Hazard Ratio (95% CI) for Mortality Associated With White Women’s Genetically Determined Age at Menarche **eFigure 4.** Interaction Between Actual and Genetically Determined Age at Menarche on Mortality in White Women
