# Supplementary material for: Joint Associations of Actual Age and Genetically Determined Age at Menarche With Risk of Mortality
Source: JAMA Netw Open. 2021 Jun 30;4(6):e2115297. doi: 10.1001/jamanetworkopen.2021.15297 (PMC8246309; doi:10.1001/jamanetworkopen.2021.15297)
Supplement: Supplement. — eTable. Characteristics of Genetic Variants Associated With Age at Menarche in the UK Biobank eFigure 1. Multivariate Adjusted Hazard Ratio (95% CI) for Mortality Associated With Women’s Genetically Determined Age at Menarche eFigure 2. Multivariate Adjusted Hazard Ratio (95% CI) for Mortality Associated With White Women’s Actual Age at Menarche eFigure 3. Multivariate Adjusted Hazard Ratio (95% CI) for Mortality Associated With White Women’s Genetically Determined Age at Menarche eFigure 4. Interaction Between Actual and Genetically Determined Age at Menarche on Mortality in White Women [file jamanetwopen-e2115297-s001.pdf]

Supplementary Online Content

Liang Z, Ma H, Song Q, et al. Joint associations of actual age and genetically determined age at menarche with risk of mortality. *JAMA Netw Open.* 2021;4(6):e2115297. doi:10.1001/jamanetworkopen.2021.15297

eTable. Characteristics of Genetic Variants Associated With Age at Menarche in the UK Biobank
eFigure 1. Multivariate Adjusted Hazard Ratio (95% CI) for Mortality Associated With Women's Genetically Determined Age at Menarche
eFigure 2. Multivariate Adjusted Hazard Ratio (95% CI) for Mortality Associated With White Women's Actual Age at Menarche
eFigure 3. Multivariate Adjusted Hazard Ratio (95% CI) for Mortality Associated With White Women's Genetically Determined Age at Menarche
eFigure 4. Interaction Between Actual and Genetically Determined Age at Menarche on Mortality in White Women

This supplementary material has been provided by the authors to give readers additional information about their work.

© 2021 Liang Z et al. *JAMA Network Open.*

eTable. Characteristics of Genetic Variants Associated With Age at Menarche in the UK Biobank

SNP	CHR	POS	REF	A2	EAF	Beta	SE	*P* value
rs1032728	1	41679566	A	T	0.880491	0.037295	0.007282	3.91E-08
rs10874340	1	83259080	G	T	0.507511	-0.02724	0.00465	1.71E-09
rs10913213	1	176563260	C	G	0.659481	0.026534	0.004915	2.68E-09
rs11165924	1	98375448	A	G	0.666275	0.025413	0.004959	3.77E-09
rs11209943	1	72750500	A	G	0.400589	0.040395	0.004744	1.01E-21
rs113184243	1	27064382	G	T	0.919543	0.045224	0.008536	4.62E-08
rs12040029	1	213451958	C	T	0.878842	0.044123	0.007455	1.34E-09
rs17449243	1	21174429	T	C	0.861235	-0.02898	0.006724	2.28E-08
rs1973284	1	243856681	A	G	0.31178	-0.0315	0.005026	6.70E-11
rs2194899	1	165410785	A	G	0.364434	-0.06148	0.004872	6.38E-44
rs2343506	1	162892924	A	C	0.399282	-0.02927	0.004783	2.16E-10
rs2697963	1	14150740	G	T	0.156716	0.05142	0.006416	1.65E-18
rs2809969	1	199802016	T	C	0.468431	0.033243	0.00468	6.44E-14
rs301803	1	8498680	A	T	0.656024	-0.02629	0.004914	1.11E-11
rs35011283	1	42766283	C	T	0.905986	0.04648	0.008039	2.09E-08
rs4133019	1	102508093	A	C	0.462361	0.035643	0.004681	2.40E-15
rs4360524	1	151099557	A	G	0.300504	-0.02845	0.005063	9.33E-09
rs4660257	1	44148168	T	C	0.70099	0.042062	0.005079	9.12E-20
rs4889	1	204159787	G	C	0.753544	0.035826	0.005398	1.91E-13
rs506589	1	177894287	T	C	0.79182	0.068496	0.005732	1.47E-39
rs61779781	1	65934354	T	C	0.57196	-0.03549	0.004696	7.41E-14
rs643428	1	54728858	C	T	0.390953	0.02493	0.004789	1.17E-08
rs66461782	1	71533287	A	T	0.729516	-0.02674	0.005242	2.32E-09
rs7553348	1	75005067	G	A	0.43822	-0.05758	0.004695	3.16E-39
rs10170108	2	137449956	G	A	0.436408	0.030418	0.004682	3.21E-13

© 2021 Liang Z et al. *JAMA Network Open.*

rs11125803	2	25052177	C	T	0.259776	-0.03295	0.005318	6.78E-11
rs112753638	2	203199219	C	T	0.884793	0.046551	0.007276	1.01E-13
rs113467400	2	11720299	C	T	0.912031	0.046655	0.008257	1.86E-08
rs11893331	2	56577406	A	G	0.830787	-0.07449	0.006202	4.46E-37
rs12476703	2	200119263	T	G	0.40376	-0.04027	0.004735	4.92E-18
rs142058842	2	156621725	C	G	0.834142	-0.07287	0.006245	3.14E-38
rs1518086	2	199625393	G	A	0.357731	-0.05732	0.004872	3.80E-37
rs1526070	2	199783596	G	A	0.507079	0.036742	0.004659	1.98E-14
rs16841741	2	209637598	A	G	0.889663	-0.05602	0.007409	3.50E-15
rs1861072	2	210851634	T	C	0.599491	0.025176	0.004754	2.39E-08
rs2060199	2	166196681	T	A	0.446395	0.022891	0.004668	9.87E-09
rs2539679	2	59856713	T	C	0.187506	-0.04218	0.005959	1.48E-13
rs2679894	2	105870779	A	G	0.421922	0.05615	0.004725	4.79E-32
rs3136247	2	48013099	A	G	0.806304	-0.04133	0.005888	1.92E-12
rs338070	2	45175289	C	G	0.418424	-0.02261	0.004763	1.88E-08
rs4353638	2	61499019	A	G	0.846216	0.053703	0.006437	1.59E-18
rs4664602	2	153542585	C	T	0.172512	0.051105	0.006148	1.26E-19
rs5017302	2	631069	G	A	0.174849	0.073479	0.006172	6.13E-38
rs56277911	2	10397352	C	T	0.902348	-0.04221	0.007843	3.05E-08
rs566465	2	169705104	A	T	0.532495	-0.02252	0.004651	2.10E-08
rs57171084	2	69693747	T	C	0.817177	0.030978	0.006022	2.95E-08
rs59072247	2	73536689	C	T	0.970496	-0.1036	0.013828	3.68E-16
rs62106258	2	417167	T	C	0.951215	-0.13128	0.010779	1.68E-40
rs62178545	2	157580792	G	A	0.680358	-0.02476	0.005008	2.33E-08
rs6429912	2	142347455	A	G	0.562032	0.038014	0.004694	1.03E-17
rs6719214	2	100607421	T	C	0.851955	0.034508	0.00654	1.07E-08
rs6735626	2	213403972	G	A	0.560183	-0.02404	0.004689	1.25E-08

rs72892124	2	157134497	A	G	0.933222	0.070552	0.009311	1.94E-16
rs842545	2	184261573	T	C	0.198693	0.032146	0.005844	9.96E-13
rs13064915	3	137121243	T	C	0.67711	-0.02531	0.005002	4.03E-08
rs13322435	3	156795468	A	G	0.598365	0.035167	0.004782	1.54E-15
rs141829833	3	51776104	C	T	0.976655	-0.15387	0.015682	3.72E-25
rs1452201	3	187349009	C	T	0.449615	0.025087	0.004705	2.90E-08
rs16860342	3	185652947	C	T	0.572984	-0.03658	0.004713	6.53E-17
rs1984870	3	24715135	G	T	0.536663	-0.03777	0.004714	1.64E-15
rs2067082	3	38438182	G	C	0.440326	0.026304	0.004699	3.90E-08
rs28672845	3	88270627	A	C	0.160511	0.042323	0.006326	2.10E-14
rs35999162	3	49597230	C	G	0.692155	0.034497	0.005048	1.70E-13
rs3849531	3	128058617	A	T	0.732993	-0.04131	0.00527	1.09E-18
rs6439371	3	132610752	G	A	0.350882	0.031114	0.004889	2.04E-12
rs7433864	3	117593483	C	A	0.495818	-0.05354	0.004662	1.84E-33
rs75412999	3	29625116	T	C	0.941049	0.048165	0.010007	3.03E-08
rs7639267	3	52568805	G	T	0.441738	-0.03278	0.00469	1.95E-15
rs7643617	3	114753078	A	C	0.903253	0.048555	0.007893	1.82E-11
rs7652234	3	24190459	C	G	0.959579	-0.07859	0.011967	1.44E-12
rs815715	3	61264084	C	G	0.573577	-0.02692	0.004732	6.87E-10
rs865809	3	183997735	A	G	0.229792	-0.03746	0.005556	5.33E-12
rs9681954	3	86892364	T	C	0.384126	-0.04701	0.004792	2.37E-25
rs9756797	3	18427477	G	A	0.594217	0.029108	0.004755	2.33E-10
rs9849338	3	172126476	C	A	0.63732	0.028918	0.004881	1.10E-10
rs10857036	4	115996673	T	C	0.43743	-0.02429	0.004728	5.62E-09
rs10938397	4	45182527	A	G	0.567437	0.043732	0.004721	7.05E-22
rs115260227	4	104774698	A	G	0.987966	-0.24537	0.022321	7.39E-32
rs1347901	4	44518235	T	C	0.345299	0.028584	0.004902	1.85E-09

© 2021 Liang Z et al. *JAMA Network Open.*

rs1351623	4	104587977	C	T	0.834417	-0.04972	0.006289	1.77E-21
rs143875052	4	106150555	C	T	0.878099	0.048452	0.007133	4.81E-14
rs17464835	4	132764481	G	A	0.799123	-0.03446	0.005809	5.74E-11
rs2391504	4	130731072	A	G	0.324523	-0.02455	0.004984	3.36E-08
rs2798224	4	3267668	G	A	0.42909	-0.02943	0.004715	1.04E-10
rs28535294	4	66470543	G	A	0.676403	0.025219	0.004986	3.89E-08
rs34811474	4	25408838	G	A	0.768652	-0.03026	0.005526	4.65E-08
rs6844340	4	104267254	G	T	0.710663	-0.02464	0.005151	3.97E-08
rs7438414	4	95265822	T	C	0.381602	0.040686	0.004832	7.44E-19
rs9991479	4	28794872	T	C	0.269838	-0.03778	0.005257	9.00E-15
rs10060622	5	87929869	T	C	0.723296	0.025092	0.005227	2.35E-08
rs10063744	5	95649772	C	G	0.715382	-0.03009	0.00519	2.31E-11
rs10941225	5	34961480	T	A	0.554294	-0.0268	0.004704	2.81E-08
rs114489117	5	92948485	T	A	0.892957	0.038421	0.007561	1.35E-08
rs11738611	5	156772974	C	A	0.788536	-0.03222	0.005701	1.74E-09
rs12374521	5	147836880	C	T	0.456322	0.022733	0.004683	2.70E-08
rs12656569	5	168738591	A	C	0.203085	0.030974	0.0058	1.59E-08
rs13179411	5	133900513	G	T	0.849694	-0.07693	0.006532	1.36E-34
rs141344917	5	111134797	G	A	0.936936	0.086994	0.009794	2.21E-17
rs1618689	5	110606055	C	G	0.276541	-0.0281	0.005212	2.50E-09
rs247520	5	110876057	T	C	0.771991	0.035633	0.005624	2.67E-13
rs2861809	5	165893943	G	A	0.560482	0.026535	0.004717	4.31E-08
rs298088	5	58994526	A	G	0.268922	0.026111	0.005256	2.63E-09
rs35375873	5	43190647	G	C	0.887257	-0.04887	0.007447	2.17E-13
rs56289631	5	137018758	G	A	0.771099	-0.03051	0.005561	7.68E-12
rs7727787	5	180653039	G	A	0.865541	0.043157	0.006841	6.87E-12
rs77470973	5	52686921	T	C	0.706	0.032387	0.005154	1.31E-10

© 2021 Liang Z et al. *JAMA Network Open.*

rs80170948	5	64020316	T	G	0.957123	-0.07366	0.012033	3.17E-10
rs9312937	5	16836005	T	C	0.516772	-0.02626	0.004727	1.93E-09
rs12193842	6	84322158	A	C	0.668071	-0.03259	0.004948	2.70E-10
rs12206564	6	100987009	T	C	0.505029	0.044509	0.004661	1.70E-26
rs13199764	6	100744134	T	C	0.77315	0.036372	0.005554	3.20E-14
rs145812241	6	105118744	C	T	0.982578	0.096512	0.018432	4.36E-08
rs1844594	6	126788107	G	A	0.544801	0.049163	0.004681	4.95E-27
rs1933801	6	105365725	C	T	0.320792	0.141884	0.004989	7.55E-203
rs2206271	6	50786008	T	A	0.708338	0.036701	0.005137	8.73E-12
rs2254479	6	41831143	G	A	0.449713	0.034271	0.004676	3.92E-15
rs2764261	6	108927842	A	G	0.372206	0.032233	0.004822	2.85E-13
rs34399458	6	56848842	C	T	0.827336	0.050007	0.006151	4.58E-19
rs34613202	6	77710316	G	C	0.796159	0.060461	0.005781	1.36E-25
rs3828755	6	43115220	A	G	0.51236	0.024693	0.004683	1.45E-09
rs62415601	6	76432116	A	C	0.624687	-0.03073	0.004817	9.49E-12
rs6456198	6	170622922	C	G	0.857828	-0.0334	0.006669	1.43E-08
rs6917291	6	77160813	C	T	0.693762	0.028725	0.005054	7.02E-11
rs7452200	6	54663168	C	T	0.530352	-0.03464	0.004665	2.75E-16
rs7746459	6	105071380	T	A	0.870494	-0.04196	0.006947	3.15E-10
rs7749175	6	98770345	C	T	0.537083	-0.02586	0.004682	3.02E-08
rs78928932	6	136228617	T	C	0.923173	-0.06085	0.009178	3.27E-08
rs9295482	6	20761234	A	C	0.661089	-0.02565	0.004934	2.07E-08
rs9321106	6	128355316	A	G	0.816907	-0.03333	0.006092	2.16E-09
rs9321659	6	100116092	G	A	0.866458	-0.06434	0.006857	3.91E-23
rs9397414	6	151806283	A	G	0.686425	0.039452	0.00503	1.64E-16
rs10226266	7	32941119	A	G	0.413962	-0.02456	0.00477	1.06E-08
rs113766063	7	129989450	G	A	0.984646	0.118061	0.019261	3.19E-08

rs113772882	7	1642196	G	T	0.695245	0.033845	0.005145	1.63E-10
rs11556924	7	129663496	C	T	0.610016	-0.02634	0.004788	1.93E-09
rs13233438	7	132690684	G	C	0.570988	-0.02627	0.004717	2.60E-09
rs13233916	7	138874416	C	G	0.91002	-0.04282	0.008149	1.88E-10
rs1860826	7	2112506	G	A	0.642469	-0.02526	0.004892	2.51E-09
rs1899692	7	121978066	G	C	0.825541	-0.03531	0.006157	1.63E-09
rs35005436	7	74134911	T	C	0.840152	0.053973	0.006393	6.47E-22
rs35775580	7	130420740	A	G	0.952672	-0.06895	0.010978	2.28E-12
rs4141153	7	41467946	A	G	0.803151	-0.06207	0.005868	1.13E-31
rs56283944	7	157983840	G	A	0.66133	-0.02805	0.004928	7.59E-09
rs62468583	7	78163424	A	T	0.883035	0.03363	0.007331	4.08E-08
rs794358	7	75195089	G	A	0.558162	-0.02479	0.004866	5.32E-10
rs9640621	7	94094153	C	T	0.412834	-0.02462	0.004747	1.69E-08
rs999885	7	99701176	G	A	0.486231	-0.02628	0.004672	4.25E-09
rs11988076	8	53128629	A	G	0.834561	-0.03077	0.006282	2.86E-08
rs12550650	8	4550990	C	T	0.549712	-0.04122	0.004702	2.80E-20
rs16939126	8	76572453	T	C	0.839748	0.033348	0.00638	4.98E-10
rs17053711	8	25311269	G	A	0.737752	-0.03009	0.0053	3.97E-09
rs2688326	8	3767623	C	T	0.304727	0.036474	0.005091	8.77E-16
rs28624558	8	57802716	G	T	0.619601	0.025754	0.004844	1.40E-08
rs2953511	8	87340870	G	A	0.748459	0.03226	0.005377	4.78E-12
rs3133974	8	94746698	G	T	0.712796	0.031053	0.005167	6.85E-09
rs34726119	8	54026145	C	T	0.777998	-0.04029	0.005619	8.33E-15
rs34746747	8	78639613	T	A	0.682145	-0.033	0.005041	6.34E-12
rs349356	8	73453946	G	T	0.297412	-0.02833	0.005121	1.96E-09
rs4266671	8	4829193	A	G	0.624278	0.038842	0.004833	2.02E-18
rs62507083	8	53842780	G	T	0.879459	0.045369	0.007174	5.80E-11

© 2021 Liang Z et al. *JAMA Network Open.*

rs62520182	8	140646051	C	T	0.849681	-0.05465	0.006669	2.25E-19
rs7007681	8	77695544	G	A	0.849867	0.034974	0.006541	4.35E-08
rs7846385	8	78160179	T	C	0.715429	0.041881	0.005163	1.69E-19
rs10780649	9	86703561	T	G	0.478397	0.047846	0.004679	1.13E-24
rs10810071	9	13992263	C	T	0.747968	0.03247	0.005399	3.14E-09
rs10820310	9	105686085	A	G	0.72726	0.027473	0.005232	1.33E-08
rs10867332	9	81651533	G	C	0.806381	-0.03036	0.005921	2.08E-08
rs10908948	9	92507497	G	A	0.566141	0.023907	0.004714	3.34E-08
rs10960303	9	11839805	C	A	0.745819	0.038027	0.005349	2.66E-15
rs10978430	9	108916009	C	T	0.686393	0.101248	0.005032	1.48E-104
rs10984237	9	121568540	T	G	0.764405	-0.0338	0.005507	2.44E-11
rs11145656	9	80639295	C	T	0.534204	0.028286	0.004681	6.74E-11
rs11534296	9	83282402	G	A	0.739187	0.044252	0.005304	3.82E-17
rs1322148	9	10251492	A	T	0.297575	-0.02464	0.005092	2.10E-08
rs1329767	9	73798371	C	A	0.64319	0.037423	0.00487	8.65E-15
rs1888072	9	92205987	T	C	0.494866	0.035326	0.004692	5.08E-19
rs2152261	9	11353512	C	A	0.93244	-0.06354	0.009338	2.43E-13
rs2267958	9	131015279	G	A	0.487369	-0.02463	0.004764	1.92E-08
rs2297605	9	127255448	G	A	0.538812	-0.03965	0.004732	8.21E-19
rs2604264	9	76904587	C	G	0.263876	0.045274	0.005296	5.07E-19
rs35436838	9	77273910	T	G	0.952616	-0.06391	0.011056	1.01E-10
rs480263	9	1709382	T	G	0.353285	0.027693	0.004869	3.01E-11
rs57314245	9	111813832	G	A	0.702617	0.036167	0.005101	1.99E-14
rs58029167	9	5850375	A	G	0.726791	-0.02328	0.005243	4.61E-08
rs62539060	9	75913958	C	T	0.933308	-0.06573	0.009333	7.97E-15
rs7028185	9	120659608	A	G	0.332896	-0.02822	0.004963	3.34E-08
rs7048022	9	109609554	C	T	0.561745	-0.02899	0.004708	5.68E-13

© 2021 Liang Z et al. *JAMA Network Open.*

rs7852169	9	114318394	C	G	0.917578	-0.10751	0.008484	5.26E-42
rs913588	9	7174673	G	A	0.479987	0.038296	0.004661	9.51E-20
rs10740399	10	74060201	A	G	0.671361	0.025553	0.004964	1.88E-09
rs10786610	10	102640663	G	C	0.346465	0.027077	0.004952	7.32E-10
rs1172955	10	97877320	T	A	0.287036	0.058589	0.005159	4.38E-33
rs1874984	10	1731871	G	C	0.509899	-0.04676	0.004668	3.53E-22
rs2532718	10	117480864	C	T	0.580447	-0.02987	0.004736	6.04E-11
rs2919290	10	126738533	T	A	0.510466	0.022217	0.004688	1.41E-08
rs4021186	10	126761837	C	A	0.415849	0.025585	0.004995	3.16E-08
rs67221163	10	134297909	C	G	0.555329	-0.02392	0.004711	3.39E-08
rs7073746	10	64904071	A	G	0.529036	-0.02442	0.004682	6.91E-10
rs7077302	10	123676662	C	G	0.081186	0.049553	0.008551	1.46E-10
rs9423024	10	126862256	C	A	0.501048	-0.03855	0.004682	2.11E-18
rs1045590	11	77376806	C	A	0.802758	-0.033	0.005876	9.96E-09
rs10895141	11	101436741	A	C	0.329966	-0.04651	0.00497	7.19E-23
rs11030282	11	28361800	C	A	0.8401	-0.04762	0.006367	1.65E-14
rs11031006	11	30226528	G	A	0.856699	-0.04742	0.006677	2.62E-15
rs11039266	11	47532395	T	G	0.72191	-0.04094	0.00523	8.67E-18
rs11605776	11	13318524	C	A	0.287447	0.054298	0.005161	3.25E-31
rs11823251	11	84752914	C	G	0.739537	-0.0276	0.005324	7.14E-09
rs12360772	11	1899962	G	A	0.812042	0.037599	0.006001	3.62E-11
rs16917237	11	27702383	G	T	0.797747	-0.04393	0.005804	3.22E-16
rs16937956	11	8404501	A	G	0.651739	-0.04554	0.004915	7.67E-24
rs17854357	11	65601560	G	C	0.833952	0.033369	0.006253	5.57E-09
rs2256464	11	77917305	C	A	0.844801	-0.03854	0.006448	9.76E-12
rs4073513	11	46071253	A	G	0.289468	-0.03126	0.00513	3.84E-12
rs4752978	11	47324862	G	A	0.824572	-0.0313	0.006161	2.82E-08

© 2021 Liang Z et al. *JAMA Network Open.*

rs4755720	11	43628749	C	T	0.392516	-0.03682	0.004795	7.42E-17
rs545075	11	94071678	G	C	0.051912	-0.06602	0.010503	3.44E-11
rs568442	11	55309822	G	A	0.672087	0.030005	0.005477	1.28E-11
rs61371450	11	16786905	G	A	0.824175	0.044769	0.006141	4.70E-15
rs7102513	11	86717987	T	C	0.218879	-0.03269	0.005746	1.82E-09
rs7115813	11	122832074	A	G	0.447345	-0.06645	0.0047	8.61E-51
rs72992070	11	115024690	A	C	0.740631	-0.03622	0.005313	1.25E-12
rs7942122	11	122729107	T	C	0.851557	-0.04367	0.006579	1.34E-10
rs7943688	11	45446724	G	T	0.694729	-0.02999	0.005072	4.61E-10
rs7949608	11	63595925	A	G	0.426871	0.024416	0.004708	2.74E-09
rs9795476	11	235894	T	G	0.246141	0.027222	0.005419	2.16E-09
rs1054442	12	49389320	A	C	0.625149	0.034126	0.004826	1.15E-15
rs10860153	12	97541532	A	G	0.665575	0.03418	0.004982	4.22E-11
rs10861880	12	108611474	T	C	0.498061	0.024106	0.004667	1.59E-08
rs10870546	12	133054609	A	G	0.278728	0.028107	0.005227	3.68E-08
rs10902475	12	132410245	C	A	0.261352	-0.02801	0.005324	2.49E-09
rs11047447	12	24601048	A	G	0.663084	-0.03343	0.004935	5.18E-12
rs112335739	12	47844662	C	T	0.894656	-0.04664	0.007597	3.43E-10
rs1131017	12	56435929	C	G	0.429859	0.020686	0.004716	4.60E-08
rs141773786	12	57475601	A	G	0.894062	-0.04406	0.007621	1.30E-08
rs7135337	12	121404155	A	C	0.408868	-0.02561	0.004755	2.01E-09
rs76369685	12	97850590	G	A	0.939703	-0.04957	0.009846	5.39E-09
rs76439972	12	92456577	C	T	0.927974	-0.04592	0.009079	3.78E-08
rs7973253	12	109878115	A	G	0.634964	-0.02413	0.004851	2.48E-08
rs11619721	13	112082513	G	T	0.908412	0.04864	0.008192	1.83E-09
rs12019957	13	97968001	T	C	0.820945	0.036648	0.006125	1.15E-08
rs1327942	13	59833110	T	A	0.569544	0.024998	0.004756	6.47E-09

rs1933437	13	28624294	G	A	0.372456	-0.03104	0.004836	1.10E-09
rs3829388	13	61984781	T	C	0.688573	-0.03062	0.00506	2.28E-10
rs7329599	13	74561706	C	T	0.236199	-0.03612	0.005508	4.70E-12
rs7992832	13	28013501	C	T	0.723019	-0.02651	0.005235	1.17E-09
rs7995668	13	74705179	A	C	0.88474	0.045472	0.007392	3.15E-10
rs803821	13	71559009	T	C	0.6623	-0.02887	0.004966	1.72E-10
rs9517913	13	100564508	G	A	0.576687	-0.03227	0.004767	2.78E-11
rs9554311	13	28867268	C	T	0.333263	-0.03089	0.004973	2.42E-10
rs9560114	13	112187882	A	T	0.734347	-0.03449	0.005328	1.46E-09
rs9568125	13	49476315	C	T	0.845314	-0.04165	0.006464	2.52E-12
rs9603605	13	40308307	T	C	0.651204	-0.03394	0.004922	3.11E-14
rs10136330	14	30514335	C	T	0.960474	0.074198	0.012034	1.08E-11
rs10150590	14	40925881	A	C	0.399701	-0.02676	0.00479	4.73E-08
rs12433775	14	37003838	C	T	0.932559	0.053576	0.009352	3.99E-08
rs12879819	14	78424783	C	T	0.48312	0.028454	0.004704	8.86E-11
rs1555406	14	101204235	C	T	0.882778	0.043147	0.007283	3.39E-10
rs4365199	14	60994213	T	G	0.302052	0.051017	0.005086	7.25E-26
rs55658396	14	97773547	G	T	0.948503	0.068902	0.010737	4.29E-11
rs6575806	14	101353211	C	A	0.277078	0.033021	0.005493	5.28E-10
rs7161194	14	101529005	A	G	0.335344	-0.03532	0.005174	5.50E-13
rs78352137	14	100920416	T	C	0.888888	0.081251	0.007439	7.07E-30
rs8007087	14	93918885	C	T	0.312252	0.03375	0.005051	3.32E-13
rs11071027	15	54364761	A	C	0.378892	0.024665	0.00483	1.13E-08
rs111491984	15	82488239	A	T	0.734708	-0.03139	0.005297	3.22E-10
rs112036939	15	41247859	A	T	0.968691	-0.07021	0.013415	1.01E-08
rs13835	15	89056040	C	A	0.575867	0.046994	0.004733	2.20E-28
rs3743266	15	60781513	T	C	0.665848	0.046669	0.004968	1.27E-23

rs3784710	15	68072458	T	C	0.773089	-0.04154	0.005588	7.25E-17
rs4243084	15	78911672	G	C	0.664572	0.025925	0.00495	2.99E-08
rs561821	15	41427864	C	T	0.622596	0.034918	0.004828	2.30E-16
rs5742915	15	74336633	T	C	0.536826	-0.03577	0.00469	6.43E-17
rs62009090	15	77837055	T	G	0.299785	0.02815	0.00511	7.43E-10
rs62023121	15	93459453	C	T	0.844932	0.040194	0.006478	6.03E-11
rs6576457	15	23809876	G	A	0.695317	0.046299	0.005093	8.38E-20
rs72741332	15	64744790	T	C	0.936768	0.0542	0.009604	1.47E-09
rs72767997	15	99288741	T	C	0.971707	-0.07762	0.014317	2.23E-10
rs75389281	15	24159672	G	C	0.893199	0.052439	0.007577	1.08E-11
rs8032760	15	47994054	T	C	0.518293	-0.02317	0.004697	9.11E-09
rs112927956	16	24724001	C	T	0.958434	-0.07314	0.011836	1.31E-09
rs114285994	16	19935763	G	A	0.861412	-0.0543	0.006772	2.32E-18
rs11642015	16	53802494	C	T	0.599682	0.07438	0.004765	3.73E-58
rs12926791	16	69849546	C	G	0.583121	-0.05799	0.004749	3.15E-37
rs12933772	16	6560430	C	G	0.682819	0.024958	0.005034	9.88E-09
rs1362376	16	52291939	T	C	0.809413	0.046476	0.005947	1.09E-16
rs139309976	16	70785409	G	A	0.987501	-0.10804	0.021091	4.51E-08
rs1704528	16	14388750	T	C	0.668758	-0.05341	0.004978	2.85E-33
rs4786084	16	6172773	G	A	0.408427	-0.02616	0.004766	3.59E-09
rs57149692	16	30142021	G	C	0.603425	-0.03211	0.004789	7.97E-13
rs7199766	16	3637429	G	A	0.794635	0.026428	0.005785	2.14E-08
rs9652589	16	20370816	C	T	0.484841	-0.02713	0.004667	2.09E-10
rs12603280	17	6034754	G	A	0.755533	0.034621	0.005484	1.92E-12
rs12603355	17	7733037	C	T	0.706918	-0.02711	0.00513	1.85E-10
rs1285245	17	77796889	G	C	0.624265	0.028008	0.004832	1.40E-09
rs17817628	17	53174727	A	G	0.713172	0.041658	0.005176	6.16E-18

© 2021 Liang Z et al. *JAMA Network Open.*

rs17817995	17	53412594	G	C	0.680411	-0.02855	0.005063	6.14E-10
rs241036	17	43731719	A	C	0.773222	-0.03689	0.005593	2.15E-13
rs2725405	17	79220224	G	C	0.558213	0.033323	0.00472	2.97E-12
rs34768269	17	77952751	T	C	0.807363	0.033363	0.005932	6.76E-10
rs58418838	17	78700955	C	T	0.650993	0.028163	0.004899	7.23E-10
rs9635759	17	49613785	G	A	0.696577	-0.07127	0.005186	1.63E-48
rs12607857	18	53213326	G	A	0.921703	-0.05258	0.008713	2.57E-10
rs12607903	18	3817134	C	T	0.278956	0.050975	0.005227	1.09E-26
rs2668767	18	44785302	T	C	0.429567	-0.05407	0.004733	4.89E-36
rs72661148	18	45371695	A	G	0.955476	-0.06197	0.011354	4.94E-08
rs8087694	18	62339300	G	C	0.194886	-0.03079	0.005923	1.50E-08
rs9945304	18	31703064	C	G	0.419982	0.027377	0.004747	9.71E-10
rs9957921	18	57891072	G	C	0.928744	-0.04799	0.009134	4.85E-09
rs10401175	19	18230274	C	A	0.460306	-0.02713	0.004694	1.85E-09
rs12462111	19	49171306	C	T	0.535825	-0.03215	0.004764	1.93E-10
rs16992771	19	5046070	C	T	0.657339	-0.03005	0.004926	1.25E-11
rs2003476	19	18806668	T	C	0.592968	-0.03035	0.004778	2.78E-11
rs3115524	19	13021359	G	A	0.383182	0.024562	0.004805	2.34E-08
rs35917007	19	1849147	A	G	0.465238	-0.04276	0.004673	1.39E-23
rs3810291	19	47569003	G	A	0.321917	0.041086	0.005005	1.81E-19
rs484353	19	7891767	G	A	0.444892	-0.03043	0.004712	2.52E-11
rs55939240	19	36185103	A	G	0.530244	-0.0342	0.004685	1.54E-13
rs61744130	19	54080067	T	C	0.951292	0.063238	0.010849	4.55E-09
rs8112411	19	9991735	G	A	0.374216	-0.05243	0.004827	2.23E-31
rs882609	19	58991479	G	A	0.545218	-0.02307	0.004705	2.83E-09
rs2425670	20	43521729	G	A	0.436337	-0.02384	0.004721	2.49E-08
rs35255016	20	37294739	C	T	0.760695	-0.03524	0.005487	5.80E-13

© 2021 Liang Z et al. *JAMA Network Open.*

rs3746619	20	54823805	C	A	0.91676	-0.05261	0.008456	8.26E-11
rs3828002	20	19702049	G	A	0.452428	-0.03	0.004744	5.49E-11
rs6010651	20	62418243	A	C	0.619292	-0.02799	0.004843	8.41E-09
rs6039251	20	8729496	T	C	0.632004	-0.02459	0.004844	1.17E-08
rs6045400	20	18421988	A	G	0.202791	0.035035	0.005829	2.95E-11
rs6046818	20	20346178	T	C	0.845187	0.038289	0.006497	9.75E-10
rs6066454	20	46364282	C	T	0.633005	-0.02611	0.004875	4.99E-08
rs6087709	20	34017944	C	G	0.710235	0.026524	0.005169	9.44E-09
rs6136033	20	17208504	A	C	0.425193	0.027926	0.004792	1.19E-08
rs7273470	20	33456921	G	C	0.759889	-0.03461	0.005482	1.37E-12
rs73898513	20	15806210	C	T	0.876222	0.038639	0.007223	2.83E-08
rs852033	20	17085493	A	C	0.2467	-0.04201	0.005431	2.56E-16
rs35184820	21	35320941	T	G	0.492016	0.031847	0.004691	1.69E-11
rs4818008	21	40611442	T	A	0.645349	0.050239	0.004898	3.00E-27
rs73204208	21	37791466	T	G	0.870359	-0.04829	0.007004	3.38E-13
rs12167144	22	31298208	A	C	0.715331	0.032565	0.00519	1.87E-12
rs5756052	22	22268759	A	G	0.519146	0.025152	0.004705	1.01E-10
rs9616551	22	49680429	A	G	0.19899	0.032652	0.005896	5.63E-10

eFigure 1. Multivariate Adjusted Hazard Ratio (95% CI) for Mortality Associated With Women's Genetically Determined Age at Menarche

Analyses are adjusted for age, race, Townsend deprivation index, smoking status, alcohol status, physical activity, menopause status, parity, BMI, healthy diet score, birth weight, assessment center, history of cancer, diabetes, CVD, hypertension and high cholesterol, the first 5 genetic principal components and genotyping array.

Genetically determined age at menarche	No. of Women	HR(95%CI)	P-value
GRS1	41097	1.10(1.01–1.19)	.03
GRS2	41120	1.09(1.01–1.18)	.04
GRS3	41096	1.04(0.95–1.12)	.40
GRS4	41125	1.00	
GRS5	41120	1.04(0.96–1.13)	.31
GRS6	41118	1.09(1.00–1.18)	.05

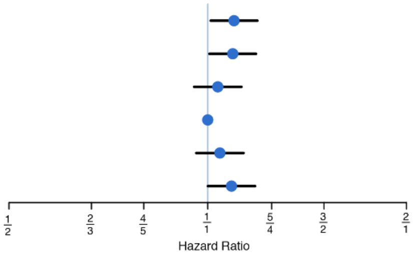

© 2021 Liang Z et al. *JAMA Network Open.*

eFigure 2. Multivariate Adjusted Hazard Ratio (95% CI) for Mortality Associated With White Women's Actual Age at Menarche

Analyses are adjusted for age, Townsend deprivation index, smoking status, alcohol status, physical activity, menopause status, parity, BMI, healthy diet score, birth weight, assessment center, history of cancer, diabetes, CVD, hypertension and high cholesterol.

White women

Actual age at menarche	No. of Women	HR(95%CI)	P-value
<12	50445	1.16(1.06–1.26)	<.001
12	47418	1.08(0.99–1.18)	.08
13	61201	1.04(0.96–1.13)	.36
14	49616	1.01(0.93–1.10)	.80
15	27355	1.00	
≥16	14091	1.19(1.06–1.33)	.002

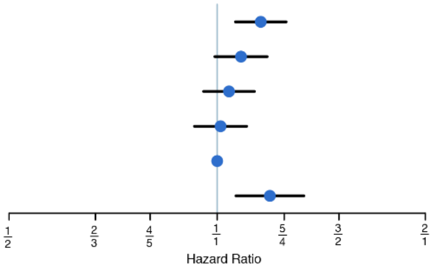

© 2021 Liang Z et al. *JAMA Network Open.*

eFigure 3. Multivariate Adjusted Hazard Ratio (95% CI) for Mortality Associated With White Women's Genetically Determined Age at Menarche
Analyses are adjusted for age, Townsend deprivation index, smoking status, alcohol status, physical activity, menopause status, parity, BMI, healthy diet score, birth weight, assessment center, history of cancer, diabetes, CVD, hypertension and high cholesterol, the first 5 genetic principal components and genotyping array.

White women

Genetically determined age at menarche	No. of Women	HR(95%CI)	P-value
GRS1	38853	1.10(1.01–1.19)	.03
GRS2	39066	1.09(1.00–1.19)	.04
GRS3	38975	1.04(0.95–1.13)	.41
GRS4	38929	1.00	
GRS5	38699	1.03(0.95–1.12)	.47
GRS6	38615	1.09(1.00–1.18)	.05

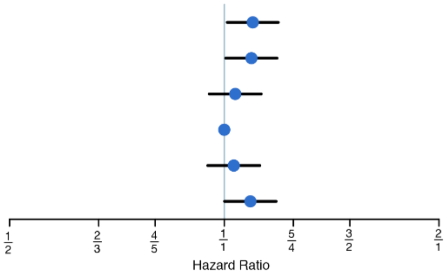

© 2021 Liang Z et al. *JAMA Network Open.*

eFigure 4. Interaction Between Actual and Genetically Determined Age at Menarche on Mortality in White Women

Analyses are adjusted for age, Townsend deprivation index, smoking status, alcohol status, physical activity, menopause status, parity, BMI, healthy diet score, birth weight, assessment center, history of cancer, diabetes, CVD, hypertension and high cholesterol, the first 5 genetic principal components and genotyping array.

Actual age at menarche	HR(95%CI)	P-value
**GRS1**		
<12	1.23(1.09–1.39)	<.001
12–13	1.12(0.99–1.25)	.06
14–15	1.08(0.93–1.26)	.30
⩾16	2.12(1.56–2.89)	<.001
**GRS2–3**		
<12	1.17(1.05–1.31)	.006
12–13	1.09(0.99–1.20)	.08
14–15	1.14(1.03–1.27)	.01
⩾16	1.37(1.12–1.67)	.001
**GRS4–5**		
<12	1.17(1.03–1.32)	.01
12–13	1.13(1.02–1.24)	.02
14–15	1.00	
⩾16	1.04(0.87–1.25)	.64
**GRS6**		
<12	1.46(1.22–1.74)	<.001
12–13	1.17(1.03–1.32)	.01
14–15	1.04(0.92–1.17)	.56
⩾16	1.34(1.12–1.60)	.001

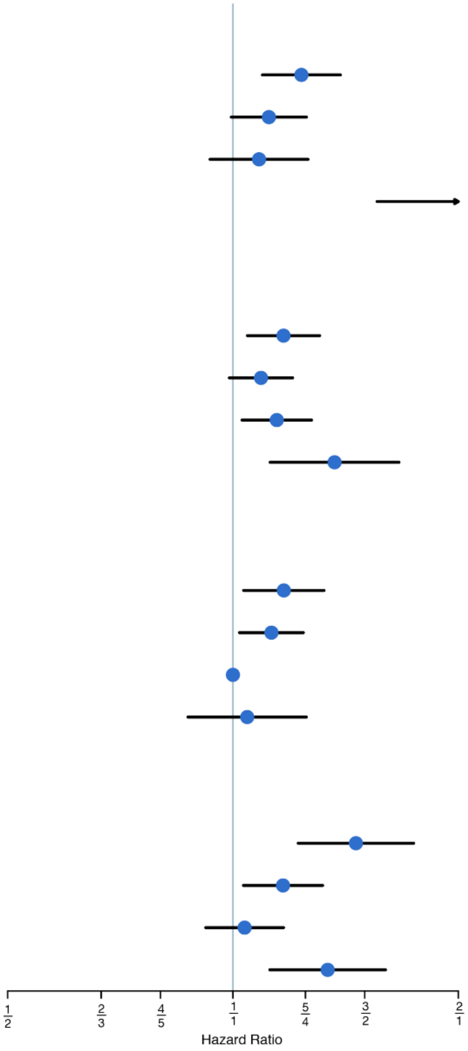

© 2021 Liang Z et al. *JAMA Network Open.*
